# Effects of lactic acid bacteria inoculation in pre-harvesting period on fermentation and feed quality properties of alfalfa silage

**DOI:** 10.5713/ajas.18.0801

**Published:** 2019-04-15

**Authors:** İbrahim Ertekin, Mustafa Kızılşimşek

**Affiliations:** 1 Department of Field Crops, Faculty of Agriculture, Hatay Mustafa Kemal University, Hatay, 31060, Turkey; 2 Department of Field Crops, Faculty of Agriculture, Kahramanmaraş Sütçü İmam University, Kahramanmaraş, 46040, Turkey

**Keywords:** Alfalfa Silage, Feed Quality, Fermentation Characteristics, Inoculation, Lactic Acid Bacteria

## Abstract

**Objective:**

To develop the fermentation quality and chemical composition of alfalfa (*Medicago sativa* Lam.) silage, plants were inoculated with different lactic acid bacteria (LAB) strains at field 24 hours before harvest.

**Methods:**

The treatment groups were as follow: silage without additive as a control and inoculated with each strains of *Lactobacillus brevis* (LS-55-2-2), *Leuconostoc citerum* (*L. citerum*; L-70-6-1), *Lactobacillus bifermentans* (*L. bifermentans*; LS-65-2-1), *Lactobacillus plantarum* (*L. plantarum*; LS-3-3) and *L. plantarum* (LS-72-2). All the silages were stored at 25°C. Parameters such as pH, microorganism and volatile fatty acid contents, crude protein, neutral detergent fiber, acid detergent fiber, net gas, metabolizable energy, organic matter digestibility, dry matter intake and relative feed value were measured to determine fermentation quality, chemical compositions and relative feed value of alfalfa silages.

**Results:**

Significant differences were found among the control and treated groups in terms of pH and microorganism contents at all opening times and crude protein, net gas, metabolizable energy and organic matter digestibility of final silage. The pH values ranged from 4.70 to 5.52 for all treatments and control silage had the highest value of overall treatments at T_75_d silages. Volatile fatty acid of silages was not influenced significantly by inoculations. However, lactic acid content of *L. bifermentans* (LS-65-2-1) was higher than the other treatments. The highest metabolizable energy and organic matter digestibility were recorded from *L. citerum* (L-70-6-1) inoculation. In addition, no significant differences were found among treatments in terms of neutral detergent fiber, acid detergent fiber, dry matter intake and relative feed value.

**Conclusion:**

Among the treated LAB isolates, *L. bifermentans* came into prominence especially in terms of organic acid composition and quality characters of silages.

## INTRODUCTION

Alfalfa, the most cultivated forage in Turkey as well as in the world, is a very substantial leguminous forage crop, grown in warm-season belt and better in quality than grass forage plants. Since alfalfa is a perennial forage crop, it can be mowed more than once in a year and generally used as dry herbage for ruminants. However, farmers have usually faced some drying problems for the first or last cutting of the alfalfa because of possible precipitation [1]. Furthermore, nutrient loss may rise to 30% under bad conditions resulting serious economic loses. Ensiling alfalfa may be a good practice in terms of eliminating the necessity of drying fresh forage and preventing nutrient loses. However, alfalfa is difficult to ensile because of its buffering capacity preventing pH drop [2–4], lack of water soluble carbohydrate (WSC) necessary for microbial proliferation during fermentation [2,5], and its relatively low dry matter (DM) content at harvest. The DM and water-soluble carbohydrate (WSC) is low and the buffering capacity is high, which prevents the pH value from falling as much as possible during the ensiling process [6–9]. One of the most important issues in silage production is to reduce pH value as fast as possible [10]. Silage fermentation is created with the supply of media acidity by using WSC via epiphytic bacteria under anaerobic conditions [11]. It is reported that the epiphytic lactic acid bacteria (LAB) on the fresh forages may be inadequate therefore rapid and sufficient pH drop may not be achieved [6,12]. Hence, LAB inoculation before ensiling the forages could help improving silage fermentation profile and enhancing feed quality [13,14]. Bacterial treatments for improving silage quality were usually applied on silage material at post-harvesting and pre-ensiling period in the previous studies, however experiments on inoculation at the pre-harvesting period in the field treatments are rare. Therefore, this study was carried out to determine the effects of pre-harvest inoculation of new LAB strains on alfalfa silage fermentation profile and feed quality.

## MATERIALS AND METHODS

### Plant, bacteria and rumen fluid providing animals

Alfalfa, “Elci” cultivar, obtained from the farmer’s field as second cut was inoculated with LAB in pre-harvesting period (24 before hours harvest). The LAB strains used as inoculant and their strain numbers as follows; *Leuconostoc citerum* (*L. citerum*; L-70-6-1), *Lactobacillus bifermentans* (*L. bifermentans*; LS-65-2-1), *Lactobacillus plantarum* (*L. plantarum*; LS-3-3) *L. plantarum* (LS-72-2) and *Lactobacillus brevis* (*L. brevis*; LS-55-2-2). *L. brevis* strain is heterofermentative while the others are homofermentative. All the strains were isolated from Turkey’s rangeland flora within the scope of a project supported by Turkish Scientific and Technical Research Organization (TUBITAK). The isolates were selected among 695 isolates considering their acid production capacity in a given time period (data not given).

To apply the *in vitro* gas production technique, three Holstein cows were used as animal donors to provide rumen fluid. The cows were fed on ration based on corn silage and intensive feed mix (19% crude protein, 2,850 kcal/kg DM). Roughage and intensive feed rate is 1/1 based on dry matter and rumen fluid was collected via rumen fistula.

### Silage preparation

Alfalfa at the 50% flowering period (DM about 370 g/kg) was theoretically inoculated with a density of 10^5^ cfu/g fresh weight with 5 bacterial inoculants prior to 24 hours from cutting on the field. Field inoculations were made at 12 m^2^ parcels with three replications and 3 m of interspace were given between each application in order to prevent any contamination. Fresh forage yield was estimated by cutting 1 m^2^ of alfalfa and weighted just before inoculation and application dosages were calculated and inoculation was done by spraying with an atomizer. A day after inoculation on the field, crops were cut by hand and chopped in 2 to 3 cm lengths via lab-type chopping machine. The machine was cleaned by 70% alcohol after chopping each treatment in order to prevent any contamination. Chopped forage materials were ensiled into plastic vacuum-package with 400±40 g with three replications for all opening times by means of vacuum sealer which removed 99.9% of O_2_ from the silage bag.

To monitor the microbial growth and pH changes for the first 48 hours of ensiling, silages were opened at T_0_ (just before ensiling), T_6_ (six hours after ensiling), T_12_ (twelve hours after ensiling), T_24_ (twenty-four hours after ensiling), and T_48_ (forty-eight hours after ensiling). For T_0_ time, samples were taken just before ensiling. For T_6_, T_12_, T_24_, and T_48_ openings 3 vacuum silages for each application (6×4×3 = 72 silos) were made separately. For the final silage (T_75_d or 75 days after ensilage) analysis, three replicated vacuum silages containing 500±50 g of each treatment were prepared.

### Microbiological and chemical analyzes

At the times T_0_ and T_75_d, 50 g (±0.5 g) of samples were separated and incubated at 78°C for 48 hours to determine the chemical compositions of each treatment. At all opening times, 20 g of the silage sample were mixed with 180 mL Ringers solution by a hand blender for 90 seconds at high speed and then filtered with Whatman 55 paper in order to get water extract. Microorganism counts, and pH measurements were made by using water extract of samples. Appropriate 10 fold dilution series were prepared for counting microorganisms. LAB counts were made in double layer MRS (De Man, Rogosa, and Sharpe) agar under anaerobic conditions incubated at 37°C for 48 hours. Counts of mold and yeast were enumerated in malt extract agar (MEA) at 37°C for 48 hours. Numbers of enterobacteria were evaluated in violet red bile glucose agar after incubation at 33°C for 18 hours.

Crude protein content was determined according to the Kjeldahl method and crude fat content of silages was determined by extraction method [15]. Crude ash content was detected in an ash furnace by burning at 550°C for 4 hours. Cell wall components such as neutral detergent fiber (NDF) and acid detergent fiber (ADF) were evaluated with ANKOM Fiber Analyzer (ANKOM Technology Corp., Fairport, NY, USA) according to the method described by Van Soest et al [16]. The Gas Production Technique reported by Menke and Steingass [17] was used to evaluate the digestibility properties under *in vitro* conditions of silages. In this way, 100 mL special syringes (Model Fortuna, Häberle Labortechnik, Lonsee-Ettlenschieb, Germany) were used to determine the gas production of the silages. Metabolic energy level of feeds was determined according to Blümmel and Orskov [18] and organic matter digestibility was calculated according to the reported method by Menke et al [19].

$$\begin{array}{l} \text{Metabolizable energy }(\text{ME},\text{MJ}/\text{kg DM})\\ =2.20+0.136\times \text{GP}+0.0057\times \text{CP}+0.00029\times {\text{CF}}^{2}\\ \text{Organic matter digestibility }(\text{OMD }\%)\\ =14.88+0.8893\times \text{GP}+0.0448\times \text{CP}+0.0651\times \text{CA} \end{array}$$

Where; GP, amount of gas produced (mL/200 mg) after 24 hours fermentation; CP, crude protein content of silage (g/kg DM); CF, crude fat content of silage (g/kg DM); CA, crude ash content of silage (g/kg DM).

Dry matter digestibility (DMD), dry matter intake (DMI), and relative feed value (RFV) of silages were calculated by the following formulas developed by Van Dyke and Anderson [20]. DMD values were used to calculate the RFV.

$$\begin{array}{l} \text{DMD }(\%)=88.9-(0.779\times \text{ADF }\%)\\ \text{DMI }(\%)=120/\text{NDF }\%\\ \text{RFV}=\text{DMD }\%\times \text{DMI }\%\times 0.775 \end{array}$$

Lactic acid (LA), volatile fatty acids (VFA) (acetic acid [AA], butyric acid [BA], and propionic acid [PA]) and ethanol (ETOH) content of T_75_d silages were made by using high pressure liquid chromatography at 42°C, 0.6 mL/min flow rate and by using refractive index detector described by Quiros et al [21] after the sample cleaning procedure.

### Statistical analyzes

The statistical evaluation of the data obtained in this study was made using the statistics program of MINITAB 17. The general linear model was used to determine the differences among the means and the Tukey pairwise (p<0.05) test was used to determine the significance level of the differences observed.

## RESULTS

### Chemical composition of alfalfa before the ensiling

The mean values and statistical groups CA, CP, NDF, ADF, and pH of the alfalfa at the time T0 are given in Table 1. The beginning CA values ranged from 80.02 to 86.16 g/kg DM, while the CP content was between 182.53 and 209.74 g/kg DM. NDF content values changed between 453.01 and 502.54 g/kg DM, while ADF values ranged from 276.89 to 311.90 g/kg DM. However, these differences among mean values of CA, NDF, and ADF contents were not statistically significant. Differences among initial pH and CP content of alfalfa forage were statistically significant even though they were very close to each other ranging from 5.99 to 6.10 and 182.53 and 209.74 g/kg DM, respectively.

### pH changing of silages

As it is seen in Table 2, the pH values at T0 opening time were around 6.07 for all treatments and decreased progressively from T_0_ to T_75_d. The lowest pH values were recorded from *L. plantarum* LS-72-2 treatment at the T_12_ and T_24_. Also, pH value of *L. plantarum* LS-72-2 was similar with *L. bifermentans* and *L. plantarum* LS-3-3 at the T_24_. *L. bifermentans* (LS-65-2-1), *L. plantarum* (LS-3-3) and *L. plantarum* (LS-72-2) treatments gave the lowest pH values for T_48_ with 5.08 which is the same for all three treatments. The pH value of control treatment for T_75_d was 5.52 which is statistically higher than all inoculant treatments. All inoculant treatments were statistically similar in terms of pH values at T_75_d opening time and all values, except *L. plantarum* LS-3-3, were below the level of 5.00.

### Microorganism changing of silages

The changes in the number of enterobacteria, LAB, yeast and mold in the silages from the beginning to resulting silage are given in Tables 3, 4. The microorganism contents of silages from T_0_ to T_75_d were highly variable. Enterobacteria, yeast and mold that are harmful for silage quality first increased till T_6_ opening times, then decreased dramatically for T_75_d silages. The number of enterobacteria in silages was generally higher in control treatment than inoculated silages at all opening times. Number of enterobacteria at T_48_ was higher than that of T_0_ meaning that they increased in the earlier phases of fermentation although enterobacteria counts were lower more than thousand times at T_75_d openings compared to T_48_ counts. Numbers of enterobacteria ranged between 1.96 and 3.71 log_10_ cfu/g for *L. brevis* (LS-55-2-2) and *L. plantarum* (LS-72-2), at the time T_75_d while their counts at T_48_ were 6.79 and 6.58 log_10_ cfu/g, respectively.

The LAB, that are very significant for silage quality, in creased in silos from T_0_ to T_75_d silage. Numbers of LAB ranged between 3.99 and 2.42 log_10_ cfu/g at T_0_ and 6.11 and 7.35 log_10_ cfu/g for *L. bifermentans* (LS-65-2-2) and control silage, respectively at T_75_d opening time. Number of molds linearly dropped from the beginning to the end of ensiling period. Yeast counts decreased 10 to 100 times at T_48_ compared to T_0_ samples and they were undetectable at T_75_d openings.

### Volatile fatty acid contents of T_75_d silages

The LA, VFA such as AA, BA, and PA and ETOH contents of alfalfa silages in the different experimental treatments at the time T_75_d are given at Table 5.

LA content of silages changed between 0.659% in fresh mat ter from *L. plantarum* (LS-3-3) and 1.484% fresh matter from *L. bifermentans* (LS-65-2-1). Only *L. citerum* (LS-70-6-1) and *L. bifermentans* (LS-65-2-1) treatments produced more LA than control treatment while other inoculant treatments produced less LA compared to control. *L. citerum* (LS-70-6-1) and *L. plantarum* (LS-3-3) produced the lowest PA and BA, respectively, which are unwanted fermentation products for silages. No ETOH content in any silage could be detected.

### Chemical composition and feed quality values of T_75_d silages

Chemical composition and feed quality values of T_75_d alfalfa silages inoculated with different bacterial isolates are shown in Tables 6, 7.

In terms of CP, *L. plantarum* (LS-72-2) treatment gave the highest values with 205.07 g/kg DM while the lowest CP value was obtained from *L. plantarum* (LS-3-3) treatment with 166.19 g/kg DM. NDF, ADF, DMI, and RFV were not affected significantly by the treatments. The highest ME and OMD were obtained from *L. citerum* (LS-70-6-1) with 9.55 MJ/kg DM and 57.33%, respectively, while the lowest values were 8.76 MJ/kg DM from *L. plantarum* (LS-3-3), 4.39 MJ/kg DM and 52.25% from *L. plantarum* (LS-72-2), respectively. Although the differences among treatments in terms of RFV, the highest value was 118.18 from *L. bifermentans* (LS-65-2-1) treatment and the lowest was 104.96 from *L. brevis* (LS-55-2-2) treatment.

## DISCUSSION

Some cell wall components such as NDF and ADF contents of alfalfa forage just before ensiling were similar to previously reported figures [22–25] meaning that they were within normal range. The pH value which is considered as a very important indicator for estimating fermentation profile and extent of fermentation quality of ensiled materials [26] were constant for all treatment at T0 as expected (Table 1). The pH values of control silages were generally higher than that of inoculated silages, indicating that inoculating in pre-harvesting period can induce a better fermentation compared to uninoculated silages. It is generally desired that the pH value be around 3.8 to 4.2 for any quality silage but this is usually not possible for silages from legumes. It is difficult to bring the pH below 5 for legume silages, especially with a relatively low DM content. However, in this study, measured pH values ranged between 4.70 and 5.52 for *L. bifermentans* (LS-65-2-1) and control silage, respectively, at the opening time T_75_d. Some researchers found that inoculated alfalfa, corn or sorghum silages had lower pH values than the control groups in their studies [27–29]. It can be speculated that a low pH value of inoculated alfalfa silage may be a result the efficiency of the strain of LAB nused in the experiment. *L. bifermentans* (LS-65-2-1) was the most effective strain in dropping pH at T_75_d (Table 2).

The microbiological flora structure of alfalfa forage started to change as early as the first six hours of ensiling and it was widely altered even in the first 48 hours of ensiling except for enterobacteria. The yeasts numbers increased at T_0_ opening, then started to decrease till T_48_ opening time and they were undetectable in the T_75_d silages. The number of molds started to decrease as soon as the forage was ensiled and continued to decrease as the ensiling duration was prolonged. The LAB content of silages generally increased, from T_0_ to T_48_. After T_12_, number of LAB in the silo passed the number of enterobacteria, yeast and mold and started to dominate fermentation in the silo. For a good quality silage fermentation, numerically higher LAB, the most significant species during ensiling [14], while lower counts of enterobacteria, yeast and mold are expected. The LAB numbers ranged from 6.11 to 7.35 log_10_ cfu/g fresh silage at T_75_d in accordance with Pitt and Leibensperger [30] and Rooke [31].

The LA production in quantity and LA rate in total acid produced in silo are important parameters for evaluating feed value of silage. The LA production by a given LAB strain depends on many factors such as their number in microbiological flora [32], their proliferation rate, temperature of environment and strain’s capacity to produce LA. In addition, low LA can cause proliferation of detrimental microorganisms, which are damaging to silage quality [33,34]. Among the selected strains according to their LA production potential, *L. citerum* (LS-70-6-1) and *L. bifermentans* (LS-65-2-1) came to forefront when considered for their LA production quantity in silo and LA rate in total fermentation products. Especially *L. bifermentans* (LS-65-2-1) strain produced 41.6% more LA than control silage and 125.2% more than *L. plantarum* (LS-3-3) strain, which produced the lowest quantity of LA. Similarly, LA rate in total fermentation products of *L. bifermentans* (LS-65-2-1) strain was 39.1% while that of control silage was 25.7%, showing that *L. bifermentans* (LS-65-2-1) strain dominated fermentation in silo during ensiling. The *L. bifermentans* (LS-65-2-1) inoculation decreased BA and PA content of silage compared to control silage at 44.1% and 20.7%, respectively. Many researchers reported that the bacterial treatments increased LA and reduced AA for inoculated silages compared to uninoculated ones [27,28,35].

The CP is the critical factor affecting the quality of com mercial feeds and roughage for ruminants [34]. The CP content of alfalfa silages changed between 166.19 and 205.07 g/kg DM for T_75_d opening time. Except for *L. plantarum* (LS-3-3) treatment, higher CP content was achieved from all of the bacterial inoculation. Especially *L. plantarum* (LS-72-2-2) and *L. bifermentans* (LS-65-2-1) inoculations which produced 13.5% and 19.6% higher protein content, respectively, of T_75_d silages compared to control. This may mean that protein degradation (proteolysis), which is influenced by several factors such as pH level, buffering capacity of raw material and VFA contents of silages, was less in these treatments than control silages. Gas production, which is basically the result of fermentation of carbohydrates to acetate, propionate and butyrate, while the contribution to gas production from protein fermentation is relatively small [36,37]. Since the cellulose and hemicellulose are digested to a certain extent by ruminant animals, NDF and ADF content of feeds are very important parameters affecting digestibility and feed intake by ruminants [36,37]. The NDF values obtained from this study, a mean of 50.94%, was higher than the desired rate that is around 25% to 32% reported by Tekce and Gul [38]. The lowest NDF and ADF from this study were obtained from *L. bifermentans* (LS-65-2-1) and *L. plantarum* (LS-72-2) with 493.09 g/kg DM and 333.85 g/kg DM, respectively. Inoculated silages with LAB strains didn’t affect the NDF and ADF contents statistically as reported by Przemysław et al [39], Đorđević et al [40], and Ce et al [41].

Higher intake and improved animal performance are associated with well fermented, highly digestible silages containing high concentrations of lactic acid, and low AA and lignocellulosic structure [42]. Gonzalez et al [43] reported that fermentation of soluble carbohydrates and protein in the silo might cause a lower content of digestible organic matter compared to the control. In the present study, LAB inoculants positively affected ME, OMD, DMI, and RFV parameters. When overall parameters are taken into consideration, *L. bifermentans* (LS-65-2-1) was generally the outstanding strain compared to the others.

## IMPLICATIONS

The LAB strains used in this study positively affected fermentation properties and feed quality parameters of treatments compared to control. It can be speculated that L. bifermentans (LS-65-2-1) strain was superior in terms of many parameters evaluated in the present study. The strain was isolated from the natural areas of Turkey and selected as a high LA producer strain. This strain can be registered based upon positive results obtained from current research and after the studies comparing it with commercial ones.

## Figures and Tables

**Table 1:** The chemical compositions of each treatment sample of the alfalfa used as the silage material at the time T0

Inoculants	CA (g/kg DM)	CP (g/kg DM)	NDF (g/kg DM)	ADF (g/kg DM)	pH
Control	82.44±1.47	183.29±5.59^b^	498.60±6.70	311.67±10.97	6.06±0.02^ab^
*Lactobacillus brevis* (LS-55-2-2)	81.79±1.43	182.53±4.54^b^	502.54±19.56	311.90±21.35	5.99±0.02^b^
*Lactobacillus citerum* (LS-70-6-1)	80.02±1.89	199.30±2.33^ab^	476.95±8.22	293.40±10.85	6.07±0.01^a^
*Lactobacillus bifermentans* (LS-65-2-1)	81.79±3.43	202.11±7.71^ab^	467.40±15.39	282.07±21.12	6.10±0.02^a^
*Lactobacillus plantarum* (LS-3-3)	84.16±3.92	186.76±3.73^b^	453.01±16.38	276.89±12.58	6.10±0.01^a^
*Lactobacillus plantarum* (LS-72-2)	86.16±0.98	209.74±1.78^a^	501.81±21.10	300.89±10.80	6.07±0.02^a^
Mean	82.73	193.96	483.39	296.14	6.07
p-value	ns	**	ns	ns	*

CA, crude ash; DM, dry matter; CP, crude protein; NDF, neutral detergent fiber; ADF, acid detergent fiber; pH, power of hydrogen; ns, non-significant.

a,b Mean values with diffent superscripts have significant difference.

** p<0.01;

* p<0.05.

**Table 2:** Effects of different bacterial inoculants on pH values of ensiled silages at different silage opening times

Inoculants	T_0_	T_6_	T_12_	T_24_	T_48_	T_75_d
Control	6.06±0.02^ab^	6.07±0.01^a^	6.02±0.01^a^	5.75±0.05^a^	5.93±0.03^a^	5.52±0.09^a^
*Lactobacillus brevis* (LS-55-2-2)	6.00±0.02^b^	6.00±0.02^ab^	5.82±0.01^ab^	5.40±0.03^ab^	5.22±0.04^bc^	4.83±0.02^b^
*Lactobacillus citerum* (LS-70-6-1)	6.07±0.01^a^	6.03±0.02^ab^	5.94±0.02^ab^	5.73±0.15^a^	5.36±0.07^b^	4.79±0.00^b^
*Lactobacillus bifermentans* (LS-65-2-1)	6.10±0.02^a^	6.06±0.02^a^	5.84±0.03^ab^	5.33±0.05^b^	5.08±0.03^c^	4.70±0.02^b^
*Lactobacillus plantarum* (LS-3-3)	6.10±0.01^a^	6.00±0.02^ab^	5.98±0.04^a^	5.28±0.07^b^	5.08±0.01^c^	5.00±0.05^b^
*Lactobacillus plantarum* (LS-72-2)	6.07±0.02^a^	5.97±0.01^b^	5.72±0.11^b^	5.18±0.04^b^	5.08±0.02^c^	4.92±0.15^b^
Mean	6.07	6.02	5.89	5.45	5.29	4.91
p-value	**	**	*	***	***	***

T_0_, fresh plants before ensiling; T_6_, six hours after ensiling; T_12_, twelve hours after ensiling; T_24_, twenty-four hours after ensiling; T_48_, forty-eight hours after ensiling; T_75_d, seventy-five days after ensiling.

a–c Mean values with diffent superscripts have significant difference.

* p<0.05;

** p<0.01;

*** p<0.001.

**Table 3:** Effects of different bacterial inoculants on numbers of enterobacteria and lactic acid bacteria of ensiled silages at different silage opening times

Inoculants	T_0_	T_6_	T_12_	T_24_	T_48_	T_75_d
Enterobacteria (log_10_ cfu/g in fresh matter)						
Control	5.60±0.06	7.02±0.10^a^	7.22±0.07^a^	7.71±0.09^a^	6.58±0.02^a^	3.59±0.20^a^
*Lactobacillus brevis* (LS-55-2-2)	5.63±0.07	6.77±0.10^ab^	5.51±0.51^b^	7.10±0.12^bc^	6.79±0.09^a^	3.71±0.04^a^
*Lactobacillus citerum* (LS-70-6-1)	5.20±0.21	6.32±0.05^c^	5.10±0.10^b^	7.39±0.07^ab^	6.68±0.17^a^	2.99±0.12^ab^
*Lactobacillus bifermentans* (LS-65-2-1)	4.99±0.03	6.75±0.12^ab^	5.66±0.56^b^	6.98±0.05^c^	6.72±0.03^a^	2.68±0.28^ab^
*Lactobacillus plantarum* (LS-3-3)	5.17±0.17	6.60±0.03^bc^	6.47±0.10^ab^	7.07±0.05^bc^	6.97±0.11^a^	3.33±0.41^a^
*Lactobacillus plantarum* (LS-72-2)	5.58±0.60	5.85±0.05^d^	6.33±0.13^ab^	5.92±0.08^d^	5.72±0.05^b^	1.96±0.19^b^
Mean	5.36	6.55	6.05	7.03	6.58	3.04
p-value	ns	***	**	***	***	**
Lactic acid bacteria (log_10_ cfu/g in fresh matter)						
Control	3.99±0.05^a^	7.02±0.10^a^	6.94±0.16^bc^	8.91±0.27	8.30±0.10^b^	7.35±0.06
*Lactobacillus brevis* (LS-55-2-2)	3.87±0.09^ab^	6.77±0.10^ab^	7.43±0.09^a^	9.14±0.17	9.22±0.16^ab^	7.30±0.23
*Lactobacillus citerum* (LS-70-6-1)	2.97±0.60abc	6.32±0.05^c^	6.57±0.09^cd^	8.74±0.13	10.79±0.87^a^	6.32±0.29
*Lactobacillus bifermentans* (LS-65-2-1)	2.42±0.26^c^	6.75±0.12^ab^	6.44±0.03^d^	9.10±0.08	9.48±0.07^ab^	6.11±0.50
*Lactobacillus plantarum* (LS-3-3)	2.60±0.14^bc^	6.60±0.03^bc^	7.40±0.14^ab^	8.76±0.12	9.23±0.16^ab^	7.00±0.03
*Lactobacillus plantarum* (LS-72-2)	3.23±0.10^d^	5.85±0.05^d^	7.07±0.05^ab^	8.81±0.12	9.10±0.02^ab^	6.76±0.30
Mean	3.18	6.55	6.98	8.91	9.35	6.81
p-value	**	***	***	ns	*	ns

T_0_, fresh plants before ensiling; T_6_, six hours after ensiling; T_12_, twelve hours after ensiling; T_24_, twenty-four hours after ensiling; T_48_, forty-eight hours after ensiling; T_75_d, seventy-five days after ensiling; ns, non-significant.

a–d Mean values with diffent superscripts have significant difference.

* p<0.05;

** p<0.01;

*** p<0.001.

**Table 4:** Effects of different bacterial inoculants on numbers of yeasts and molds of ensiled silages at different silage opening times

Inoculants	T_0_	T_6_	T_12_	T_24_	T_48_	T_75_d
Yeasts (log_10_ cfu/g in fresh matter)						
Control	4.36±0.18	4.73±0.13^b^	4.46±0.33^bc^	4.06±0.15^a^	3.25±0.48^a^	nd
*Lactobacillus brevis* (LS-55-2-2)	4.66±0.18	5.14±0.05^b^	5.08±0.18^bc^	2.59±0.06^b^	3.21±0.22^a^	nd
*Lactobacillus citerum* (LS-70-6-1)	3.92±0.24	6.15±0.10^a^	4.64±0.07^bc^	4.35±0.56^a^	3.37±0.25^a^	nd
*Lactobacillus bifermentans* (LS-65-2-1)	4.00±0.00	6.17±0.15^a^	5.18±0.53abc	2.26±0.14^b^	2.82±0.02^a^	nd
*Lactobacillus plantarum* (LS-3-3)	4.00±0.00	5.73±0.08^a^	6.41±0.01^a^	2.30±0.17^b^	1.26±0.14^b^	nd
*Lactobacillus plantarum* (LS-72-2)	4.01±0.23	5.04±0.17^b^	5.96±0.21^ab^	2.26±0.14^b^	2.42±0.25^ab^	nd
Mean	4.16	5.49	5.29	2.97	2.72	-
p-value	ns	***	**	***	**	-
Molds (log_10_ cfu/g in fresh matter)						
Control	4.88±0.10^a^	4.26±0.22^a^	3.84±0.23^a^	3.79±0.04^a^	3.13±0.13^ab^	1.30±0.00^c^
*Lactobacillus brevis* (LS-55-2-2)	4.93±0.24^a^	3.77±0.09^ab^	3.67±0.09^ab^	2.79±0.10^bc^	3.49±0.17^a^	1.69±0.20^bc^
*Lactobacillus citerum* (LS-70-6-1)	4.41±0.29^ab^	3.45±0.31^b^	2.59±0.06^c^	3.44±0.18^ab^	2.71±0.11^ab^	1.71±0.21abc
*Lactobacillus bifermentans* (LS-65-2-1)	3.95±0.05^b^	3.10±0.10^b^	2.59±0.15^c^	2.59±0.17^c^	2.58±0.18^bc^	2.38±0.19^a^
*Lactobacillus plantarum* (LS-3-3)	3.93±0.19^b^	3.00±0.00^b^	3.25±0.15^ab^	2.26±0.14^c^	1.65±0.35^c^	2.06±0.03^ab^
*Lactobacillus plantarum* (LS-72-2)	4.54±0.14^b^	3.70±0.10^bc^	3.05±0.03^bc^	2.70±0.20^c^	2.26±0.07^bc^	1.69±0.05^bc^
Mean	4.44	3.55	3.17	2.93	2.64	1.81
p-value	**	**	***	***	***	**

T_0_, fresh plants before ensiling; T_6_, six hours after ensiling; T_12_, twelve hours after ensiling; T_24_, twenty-four hours after ensiling; T_48_, forty-eight hours after ensiling; T_75_d, seventy-five days after ensiling; nd, not detected; ns, non-significant.

a–c Mean values with diffent superscripts have significant difference.

** p<0.01;

*** p<0.001.

**Table 5:** Effects of different bacterial inoculants on volatile fatty acid contents of T_75_d silages

Inoculants	LA	AA	BA	PA	ETOH

	-------------------------------------------------------- % fresh matter ---------------------------------------------------------				
Control	1.048±0.27	0.816±0.26	1.509±0.46	0.707±0.22	nd
*Lactobacillus brevis* (LS-55-2-2)	0.933±0.39	0.604±0.09	1.283±0.25	0.543±0.08	nd
*Lactobacillus citerum* (LS-70-6-1)	1.369±0.14	0.826±0.09	1.142±0.09	0.691±0.06	nd
*Lactobacillus bifermentans* (LS-65-2-1)	1.484±0.06	0.900±0.11	0.844±0.08	0.561±0.05	nd
*Lactobacillus plantarum* (LS-3-3)	0.659±0.25	1.250±0.30	1.171±0.13	0.754±0.13	nd
*Lactobacillus plantarum* (LS-72-2)	0.955±0.12	1.454±0.12	1.563±0.29	0.868±0.04	nd
Mean	1.075	0.975	1.252	0.687	-
p-value	ns	ns	ns	ns	-

LA, lactic acid; AA, acetic acid; BA, butyric acid; PA, propionic acid; ETOH, ethanol; nd, not detected; ns, non-significant.

**Table 6:** Effects of different bacterial inoculants on means of chemical compositions and gas production of ensiled silages opened at time T_75_d

Inoculants	CP (g/kg DM)	NG (mL/g)	NDF (g/kg DM)	ADF (g/kg DM)
Control	171.54±2.09^bc^	214.33±2.70^ab^	503.83±9.72	396.73±14.51
*Lactobacillus brevis* (LS-55-2-2)	173.48±0.31^bc^	216.60±10.35^ab^	527.62±40.22	390.01±31.31
*Lactobacillus citerum* (LS-70-6-1)	187.56±3.70abc	230.96±1.02a	523.90±18.59	347.06±10.98
*Lactobacillus bifermentans* (LS-65-2-1)	194.74±3.98^ab^	223.27±1.24^ab^	493.09±14.52	338.80±4.88
*Lactobacillus plantarum* (LS-3-3)	166.19±12.30^c^	206.26±5.36^b^	505.90±18.28	381.64±11.83
*Lactobacillus plantarum* (LS-72-2)	205.07±1.06^a^	201.76±2.34^b^	501.89±14.85	333.85±3.47
Mean	183.10	215.53	509.37	364.68
p-value	**	*	ns	ns

CP, crude protein; DM, dry matter; NG, net gas; NDF, neutral detergent fiber; ADF, acid detergent fiber; ns, non-significant.

a–c Mean values with diffent superscripts have significant difference.

* p<0.05;

** p<0.01.

**Table 7:** Effects of different bacterial inoculants on means of nutritive values of ensiled silages opened at the time T_75_d

Inoculants	ME (MJ/kg DM)	OMD (%)	DMI (%)	RFV
Control	9.01±0.08^ab^	54.26±0.46^ab^	2.38±0.05	107.09±2.21
Lactobacillus brevis (LS-55-2-2)	9.08±0.28^ab^	54.66±1.85^ab^	2.30±0.18	104.96±12.33
Lactobacillus citerum (LS-70-6-1)	9.55±0.01^a^	57.33±0.17^a^	2.30±0.08	110.18±5.14
*Lactobacillus bifermentans* (LS-65-2-1)	9.38±0.06^ab^	55.99±0.25^ab^	2.44±0.07	118.12±3.80
*Lactobacillus plantarum* (LS-3-3)	8.76±0.21^b^	52.85±0.98^b^	2.38±0.09	109.04±4.04
*Lactobacillus plantarum* (LS-72-2)	8.86±0.08^ab^	52.25±0.51^b^	2.44±0.07	116.77±3.70
Mean	9.11	54.56	2.37	111.03
p-value	*	*	ns	ns

ME, metabolizable energy; OMD, organic matter digestibility; DMI, dry matter intake; RFV, relative feed value; ns, non-significant.

a,b Mean values with diffent superscripts have significant difference.

* p<0.05.
